# The Utility of Maternal Blood S100B in Women with Suspected or Established Preeclampsia—A Systematic Review

**DOI:** 10.3390/biom15060840

**Published:** 2025-06-08

**Authors:** Grigorios Karampas, Athanasios Tzelepis, Sevasti Koulouraki, Despoina Lykou, Dimitra Metallinou, Lena Erlandsson, Konstantinos Panoulis, Nikolaos Vlahos, Stefan Rocco Hansson, Makarios Eleftheriades

**Affiliations:** 1 Second Department of Obstetrics and Gynaecology, Aretaieio University Hospital, National and Kapodistrian University of Athens, 11528 Athens, Greece; thanasistzel@yahoo.gr (A.T.); sevasti.koulouraki@gmail.com (S.K.); panouliskonstantinos@gmail.com (K.P.); nfvlahos@gmail.com (N.V.); makarios@hotmail.co.uk (M.E.); 2 Division of Obstetrics and Gynecology, Institute of Clinical Sciences Lund, Lund University, 22185 Lund, Sweden; despoina.lykou@med.lu.se (D.L.); lena.erlandsson@med.lu.se (L.E.); stefan.hansson@med.lu.se (S.R.H.); 3 Department of Obstetrics and Gynecology, Skåne University Hospital, 20502 Malmö, Sweden; 4 Department of Midwifery, School of Health and Care Sciences, University of West Attica, 12243 Athens, Greece; dmetallinou@uniwa.gr

**Keywords:** pregnancy, preeclampsia, eclampsia, S100B, blood, brain injury

## Abstract

Purpose: Preeclampsia is a serious pregnancy complication without curative treatment. The central nervous system (CNS) is affected in severe cases of preeclampsia. Until now, no biomarker or other predictive method has been established for predicting severe CNS injury, including the development of eclampsia and/or long-term complications. In this systematic review, we aimed to investigate the association between maternal blood (serum or plasma) S100B levels and preeclampsia, focusing on its predictive value and correlation with the severity of the disease, with a particular focus on neurological symptoms. Methods: A search of online databases, including Medline via PubMed, Scopus databases, and Web of Science, was performed based on the PRISMA guidelines for systematic reviews. Results: Ten case–control studies that met the inclusion criteria were identified and further evaluated according to the Newcastle–Ottawa Scale (NOS). All of the studies revealed that S100B blood levels were higher in preeclampsia compared to uncomplicated pregnancies before onset, after its diagnosis, and one year postpartum. Its predictive value seems to be adequate long before the onset of preeclampsia, especially in the early third trimester. Furthermore, its levels seem to correlate with severe complications during pregnancy, such as eclampsia and HELLP syndrome, as well as neurological dysfunction postpartum. Conclusions: S100B is a promising biomarker for the prediction of acute and long-term CNS injury in preeclampsia. Still, additional studies should be conducted in order to establish a standard method of measurement and solidify its clinical use in preeclampsia management, providing individualized care in order to improve perinatal outcomes and provide personalized follow-up postpartum.

## 1. Introduction

Preeclampsia (PE) is an intricate pregnancy-related complication that exclusively occurs in humans [1]. It is among the most severe complications of pregnancy and is a major contributor to perinatal morbidity and mortality [2,3]. Globally, an estimated 8.5 million women are diagnosed with PE, formerly known as toxemia, every year, resulting in over 76,000 maternal deaths and 500,000 infant fatalities [1,2].

Despite extensive research, PE remains an unpredictable, life-threatening disease with long- and short-term consequences for both women and infants overcoming the initial organ insult [3]. It is characterized by new onset of hypertension after 20 weeks of gestation, accompanied by at least one related maternal organ dysfunction or utero-placental insufficiency [1].

Once PE is established, it poses an increased risk of serious short- and long-term complications for the mother and the fetus [3,4]. Women who have experienced PE face diminished life expectancy and an increased risk of developing cardiovascular disease, stroke, and diabetes [3,5]. Infants born from PE pregnancies face elevated risks of preterm birth, perinatal death, neurodevelopmental delays, and future cardiovascular and metabolic disorders [3,4,5]. Currently, there is no curative therapy for PE, although novel therapeutic agents are under investigation [6,7]. Thus, symptomatic treatment and delivery of the fetus with the placenta remain the only definitive cure [1]. Given the importance of preventing PE, various predictive tools have been proposed, including assessments of maternal characteristics, different biomarkers, Doppler ultrasound parameters, or combinations of the above [8,9,10]. If screening tests indicate a high risk of developing PE, the use of oral low-dose aspirin as a prophylactic treatment is recommended [11].

Preeclamptic encephalopathy remains under investigation and is associated with increased maternal risks of short- and long-term consequences [12,13]. Acute cerebral complications during pregnancy or postpartum in women with PE include eclampsia (ECL) and ischemic or hemorrhagic stroke, both of which carry high morbidity and mortality rates [14]. Eclampsia is characterized by generalized tonic–clonic seizures, and it is linked to cerebral edema, white-matter hemorrhage, and parenchymal necrosis [15]. The underlying pathophysiology and the reason why less than 2% of women with severe PE develop ECL remain unknown [14]. Interestingly, two other neurological disorders, posterior reversible encephalopathy syndrome (PRES) and reversible cerebral vasoconstriction syndrome (RCVS), are characterized by preeclampsia-like neurological symptoms, including headaches, visual disorders, and seizures, accompanied by reversible subcortical vasogenic brain edema [16,17,18]. Typically, the symptoms resolve when the underlying cause is treated, and they may partially explain the underlying pathophysiology of central nervous system (CNS) dysfunction in women with PE [18]. Following the initial brain insult, the long-term neurological consequences of PE vary extensively. Thus, PE is recognized as a sex-specific risk factor for stroke, vascular dementia, epilepsy, and Alzheimer’s disease later in life [19,20,21,22], with the risk being higher in cases occurring before 34 weeks of gestation and those associated with greater disease severity [23].

Significant efforts have been made to identify effective methods for predicting short- and long-term CNS complications in PE. Women with a history of PE often exhibit lasting neurological impairments [24,25], including white-matter hyperintensities and brain volume changes [26,27,28], which correlate with cognitive decline [29,30]. However, imaging techniques typically detect CNS alterations either late in the course of PE or years postpartum, posing practical and cost-effectiveness challenges as routine diagnostic tools. Therefore, alternative methods for predicting and monitoring CNS dysfunction in PE are under investigation, with brain-specific injury biomarkers emerging as a promising option.

The association between S100B and neurological functions or neurodevelopmental outcomes has been extensively investigated [31,32,33]. S100B is a calcium-binding glial-specific protein expressed primarily by the astrocytes (and in lower amounts by the oligodendrocytes) in the CNS, as well as by Schwann cells in the peripheral nervous system (PNS). It plays a dual role in both the CNS and PNS, supporting neuronal growth and survival at low concentrations, but promoting inflammation and cell damage at elevated levels [34,35,36,37]. Thus, it is recognized as a sensitive biomarker for brain injury and neurodegenerative diseases, detectable even in the offspring in the first hours of neonatal life [32]. Beyond the nervous system, S100B is produced by other cells, including T and B lymphocytes, adipocytes, melanocytes, enteric glial cells, and pregnancy-specific tissues such as placental trophoblasts, the decidua, and the amniotic membranes [38,39]. The expanding understanding of S100B as a biomarker has spurred interest in its relevance to maternal health, particularly in the context of PE [33].

In light of the above, this systematic review aims to comprehensively evaluate the existing evidence regarding the association between maternal blood S100B levels, disease severity, and the prediction of severe CNS manifestations as well as long-term neurological outcomes. By synthesizing and analyzing current data, we seek to evaluate the evidence for S100B as a predictive biomarker of CNS damage, offering insights that could ultimately enhance clinical risk assessment, diagnostic accuracy, therapeutic strategies, and long-term follow-up for this complex disorder.

## 2. Methods

This systematic review was conducted following the Preferred Reporting Items for Systematic Reviews and Meta-Analyses (PRISMA) guidelines [40]. PRISMA 2020 checklists for the abstract and the manuscript are provided in Supplementary Files S1. The study review was not registered. Two independent authors (T.T. and S.K.) performed the initial search in May 2024, with an updated search performed in January 2025. After removing duplicates and excluding irrelevant studies, full-text articles were screened for eligibility. In the case of discrepancy between the two authors, the first author (K.G.) reviewed the study to reach the final decision. Data of interest were then extracted from the included studies by T.T., L.D., and K.G for final analysis.

### 2.1. Search Strategy

Studies were retrieved from the PubMed, Scopus, and Web of Science databases. The search was conducted using keywords and MeSH terms related to S100B, preeclampsia, eclampsia, and pregnancy, which were adjusted as needed to align with each database’s search engine.

### 2.2. Study Selection

For full manuscript evaluation, studies had to meet the following criteria: (a) case series or case–control studies of (b) pregnancies complicated with PE–ECL, analyzing (c) S100B levels in the plasma or serum of the studied population. Only published articles in English were included in this systematic review, which exempted the need for Institutional Review Board approval but adhered to the principles outlined in the Declaration of Helsinki. No time limitations were imposed on the publication dates of the included studies.

The selection of the studies was conducted according to the PICOS criteria [41], outlined below:

(1) Participants: Pregnancies complicated by PE and/or ECL and appropriate controls.
(2) Intervention: Measurement of maternal serum or plasma S100B levels at any stage of pregnancy or postpartum.
(3) Comparator: Levels of S100B in the serum or plasma of controls.
(4) Outcome: Development, severity, or complications related to PE and ECL.
(5) Study designs: Case series or prospective–retrospective case–control studies.

Exclusion criteria: Languages other than English, thesis documentations, studies with inaccessible full-text articles, irrelevant population or biological material used, case reports, letters to the editor, conference abstracts, and review articles.

### 2.3. Primary Outcomes

The primary outcomes were the occurrence of PE or ECL. As no limitation in time was imposed on the publication dates of the studies under evaluation, the most recent revised and wider definition for the diagnosis of PE from the International Society for the study of Hypertension in Pregnancy (ISSHP) was used [1,42]. Thus, PE was defined as the onset of hypertension after 20 weeks of gestation (specifically, a systolic blood pressure (SBP) of 140 mmHg or higher, and/or a diastolic blood pressure (DBP) of 90 mmHg or higher), along with either utero-placental or maternal end-organ dysfunctions. Women with SBP ≥ 160 and/or DBP ≥ 110 mmHg, development of HELLP (hemolysis, elevated liver enzymes, and low platelet count) syndrome, or other severe organ manifestations were mentioned as severe PE (SPE) according to the ISSHP [1]. Women with PE complicated with tonic seizures were reported as ECL [1,42].

### 2.4. Data Extraction

Every retrieved article was thoroughly reviewed, encompassing a comprehensive examination from abstract to conclusion, in order to ascertain adherence to the inclusion criteria and identify the presence of any exclusionary factors. Data extraction was performed using a structured database including multiple variables of maternal and neonatal characteristics, along with maternal S100B levels in blood samples. The first author’s name, publication year, definition of PE used, study population, ethnicity, mean maternal age and body mass index (BMI), nulliparity, pregnancy type (singleton or multiple), smoking status, prophylactic use of aspirin or magnesium sulfate (MgSO_4_) treatment during pregnancy, gestational age at delivery and neonatal birth weight (g), manifestations of SPE, sample type (plasma or serum), method of S100B measurement, sampling time during pregnancy, blood levels of S100B, *p*-values, and sensitivity/specificity/predictive values were extracted when available. Qualitative variables were reported as frequencies and/or percentages. Quantitative variables were expressed as the mean ± standard deviation (SD) for normally distributed data, and as the median with interquartile range (IQR) for skewed distributions.

### 2.5. Search Results

**Figure 1:**
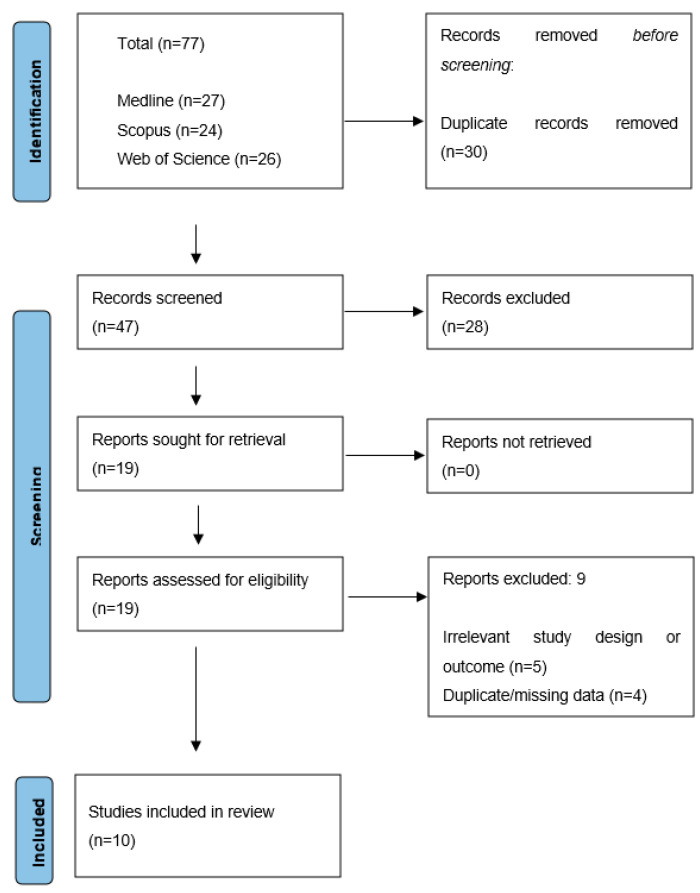
Flow diagram of study selection.

The search strategy for the identification of relevant reports yielded 77 records, which were reduced to 47 after using the deduplication function in the EndNote21.5. software (Clarivate Analytics™, London, UK). All remaining records were screened based on their title and/or abstract. Following the initial screening, 28 articles were excluded for various reasons, such as non-human study population, irrelevant study design, language other than English, use of biological fluids other than blood, and unavailability of full text for evaluation. Therefore, 19 records were considered eligible for full-text evaluation, from which an additional 9 were excluded, mainly due to irrelevant study design, outcomes, and duplicate or missing data. Ultimately, 10 studies fully met the inclusion criteria and were included in the review. Figure 1 illustrates the PRISMA flow diagram, demonstrating the process of study identification, selection, and inclusion employed in the current review.

### 2.6. Study Quality Assessment and Risk of Bias

Study quality was assessed using the Newcastle–Ottawa Quality Assessment Scale for Case–Control Studies [43] (Appendix A).

To ensure the reliability of exposure identification, measures were taken to minimize the risk of misclassification. Specifically, efforts were made to prevent the incorrect classification of cases as having PE when they did not, as well as to avoid misidentifying cases without PE as affected. This approach helped maintain the validity and accuracy of the data included in the review.

### 2.7. Characteristics of the Included Studies

#### 2.7.1. General Characteristics

The selected studies included 10 case–control studies, of which 8 were of high and 2 of low quality (Table 1) [38,44,45,46,47,48,49,50,51,52]. In total, four hundred and seventy-four (*n* = 474) individuals participated across the included studies (Table 2). Maternal and neonatal characteristics are presented in Table 2.

Six (*n* = 6) studies used the former classification for the diagnosis of PE, in which the presence of proteinuria was obligatory, while the latest four used the revised one. Three of the studies reported two groups of women with either normal (NP) or PE pregnancies, without further classification; one study reported four groups, additionally including women with pregnancy hypertension (HT) and ECL; and in the six remaining (*n* = 6) studies, PE pregnancies were divided into early/late and mild/severe groups (Table 2). Ethnicity was mentioned in only 2 studies [44,50]: in the study by Schmidt et al. [44], no significant difference was observed among the study groups regarding percentages of different races, while in the study by Wu et al., all women were of Asian origin [50].

No significant difference was observed regarding the maternal age between the NP and PE groups, apart from the studies by Schmidt et al. [44], where the ECL group included younger women compared to the other three groups, and by Vettorazzi et al. [45], which included an SPE group with women older than the other two groups. In 7 out of the 10 studies, BMI was provided, showing that the PE groups had higher BMI than the NP groups, while the percentage of nulliparous women was higher in the PE groups in all studies reporting relative data (*n* = 6). Moreover, five studies reported that only singleton pregnancies were included in the study population, while the rest did not provide any data on the number of fetuses. As expected, the length of gestation was shorter and the birth weight was lower in the PE groups in most of the studies (*n* = 7) that provided the relevant data. No significant difference was observed in smoking frequency among the different groups in any of the studies. Moreover, the prophylactic use of aspirin during pregnancy was not reported in any of the studies, while only four studies reported the use of MgSO_4_. In the study by Vettorazzi et al. [45], all women with SPE received MgSO_4_ treatment, but only eight of them were under treatment at the sampling time for S100B measurement. In the study by Artunc-Ulkumen et al. [48], blood samples for S100B assessment were obtained before the MgSO_4_ treatment started, which was administered in all women in the SPE group. On the other hand, none of the women in the study by Friis et al. needed MgSO_4_ treatment [52].

#### 2.7.2. Clinical Manifestations of Severe PE

Table 3 summarizes data regarding the criteria (symptoms, signs, or laboratory findings) for the diagnosis of SPE and their occurrence in the different study populations. As presented in Table 3, only a few studies provided detailed information on SPE criteria, especially regarding CNS symptoms/signs taken into consideration for the diagnosis. Thus, the clinical manifestations of CNS dysfunction in different studies included premonitory symptoms (such as nausea, vomiting, cephalalgia, and vision disturbances in the form of photophobia, blurry vision, diplopia, scintillating scotomas, or temporary vision loss), altered tendon reflexes, brain stroke, and ECL. In advance, even fewer studies included a separate correlation analysis on S100B levels and neurological symptoms or signs. The following manifestations were frequently reported criteria in SPE: SBP ≥ 160 mmHg and/or DBP ≥ 110 mmHg detected in two separate measurements with at least 6 h of interval, acute pulmonary edema, persistent abdominal pain in the epigastrium or the upper right quadrant, ≥5 g proteinuria in 24 h urine collection, oliguria (<500 mL/24 h) or new established renal insufficiency (serum creatinine > 1.1 mg/dL), thrombocytopenia (platelets <100.000/μL), increased hepatic enzymes (at least twice the reference values of alanine or aspartate aminotransferase), and fetal distress (including fetal growth restriction: estimated fetal weight < than the 10th centile for the specific population, oligohydramnios, abnormal Doppler ultrasound parameters of the umbilical arteries, or non-reassuring non-stress test).

#### 2.7.3. Laboratory Parameters, Blood Levels, Predictive Value of S100B, and Summary of Conclusions

Laboratory parameters, blood S100B levels measured during the gestational period and postpartum, and final statistical results were retrieved from the studies (Table 4). In addition, Table 5 summarizes the conclusions from each of the included studies.

Plasma was used for analysis in 6 of the 10 studies, and serum in the remaining 4 (Table 4). Considerable heterogeneity was observed in the methods used for laboratory analysis of S100B levels, with only 5 of the 10 studies using the same method (Sangtec 100 ELISA method, Diasorin, Stillwater, MN, USA). Of the remainder, four studies used plasma and one used serum for the analysis.

The sampling time was explicitly stated in eight (*n* = 8) studies, of which six (*n* = 6) measured S100B levels during the third trimester (Table 4). One study reported measurements taken at different gestational timepoints throughout pregnancy [46], and one measured S100B levels within the first year postpartum [49]. Although not explicitly stated, the third trimester appears to have been the sampling period in the remaining two studies [45,53].

All of the included studies consistently reported significantly higher S100B concentrations in the blood of women with various types of PE compared to those with normal pregnancies, with the highest levels observed in severe cases or those complicated by ECL (Table 4 and Table 5). Furthermore, three studies (*n* = 3) provided data on the sensitivity and specificity of specific S100B cutoff values, with the area under the curve (AUC) ranging from 0.71 to 80.7. In summary, due to the substantial heterogeneity among the included studies, a meaningful meta-analysis to assess the predictive or prognostic value of S100B could not be conducted.

#### 2.7.4. S100B Initial Detection

In detail, Schmidt et al. [44] outlined the importance of S100B as a biomarker by measuring it for the first time in different groups of pregnant women in the 3rd trimester (NP, HT, PE, and ECL). Their findings demonstrated that women suffering from ECL had elevated S100B levels compared to the other groups. In the study by Vettorazzi et al. [45], S100B was measured in the sera of three different groups, including NP, mild PE (MPE), and SPE during the 3rd trimester, highlighting that women with SPE (*n* = 34) exhibited the highest S100B levels compared to the other two groups, even after excluding five (*n* = 5) severe cases from the statistical analysis that progressed to ECL. Subgroup analysis between the rest of the women in the SPE group (*n* = 29) and women with ECL (*n* = 5) could not demonstrate a significant difference in S100B levels, indicating that changes in S100B are not dependent on the progression from SPE to ECL. In contrast, no significant difference was observed between the NP and MPE groups. Moreover, neither women with HELLP syndrome (*n* = 8) nor women with premonitory symptoms (23.5%) at the time of sampling showed significant differences in S100B levels when compared to the rest of the women with SPE, providing a correlation analysis between blood S100B levels and neurological symptoms for the first time. Similarly, within the SPE group, no differences were observed between women receiving either MgSO_4_ (*n* = 8) or antihypertensive therapy (*n* = 19; nifedipine) and those who did not require any type of therapy. Finally, women undergoing corticosteroid therapy as antenatal treatment to accelerate fetal lung maturation (*n* = 21) showed no significant differences in S100B levels compared to the rest of the study participants (*n* = 40).

#### 2.7.5. S100B During Pregnancy

Wikstrom et al. [46] measured S100B in the plasma of pregnant women at different gestational weeks (10, 25, 28, 33, and 37) in both NP and PE pregnancies, providing insights into the dynamic changes in S100B during pregnancy. In women with NP, S100B levels did not change significantly between gestational weeks 10 and 37 (0.047 vs. 0.052 μg/L; *p* = 0.71), indicating that its levels are not correlated with gestational age in uncomplicated pregnancies. On the other hand, S100B levels in the PE group began to increase several weeks before the onset of clinical manifestations, with a significant difference observed between weeks 10 and 37 (S100B levels 0.052 vs. 0.075 μg/L; *p* < 0.05). Moreover, the S100B levels did not differ between NP and PE at gestational weeks 10, 25, or 28 (*p*-values of 0.37, 0.99, and 0.60, respectively), while at weeks 33 and 37, women with PE had higher levels of S100B than controls (*p* = 0.047 and *p* = 0.01, respectively).

#### 2.7.6. S100B as a Predictive and Prognostic Biomarker

The study by Bergman et al. [47] explored the differences in the concentration of S100B levels between normal and PE pregnancies, as well as the association between clinical manifestations of PE and S100B levels. The study confirmed that plasma levels of S100B were elevated among women with PE irrespective of blood pressure levels, but in association with visual disturbances, reflecting possible CNS effects. Additionally, a cutoff value for S100B (0.14 μg/L) for the prediction of PE was introduced for the first time, with low sensitivity (44%), high specificity (86%), and an acceptable area under the curve (AUC 71.0%). Interestingly, the postpartum S100B levels in 12 women with PE were significantly higher when compared with samples collected antepartum in the same group of women (0.16 µg/L, 0.03–0.77 µg/L; vs. 0.11 µg/L, 0.02–0.33 µg/L; *p* < 0.05). Artunc-Ulkumen et al. [48] conducted the first simultaneous analysis of pregnancy-associated plasma protein A (PAPP-A), IL-6, and S100B in women with SPE and confirmed that serum S100B is elevated in cases of SPE, presenting a different cutoff value (0.0975 μg/L) with higher sensitivity (81.4%), lower specificity (58.3%), a similar AUC (71.2%), and positive/negative predictive values of 59.45% and 80.7%, respectively. The discrepancy between those two studies regarding sensitivity and specificity could be attributed to the fact that the study by Artunc-Ulkumen B. et al. [48] included only women with SPE, who typically have higher S100B concentrations than women with PE in general [48]. Moreover, in the same study, S100B levels above the cutoff value had a 12.75-fold increased risk for CNS symptoms (OR 12.75; 95% CI 2.69–60.28) and a 3.27-fold increased risk for HELLP syndrome (OR 3.27; 95% CI 0.62–17.36), highlighting the prognostic value of elevated S100B concentrations for disease severity. Additionally, for the first time, elevated levels of S100B were correlated with reduced levels of IL-6, although the underlying pathophysiology of this relationship remains unclear.

#### 2.7.7. Postpartum S100B Levels

An interesting dimension of the dynamic changes in S100B levels in PE was provided by Bergman et al. [49], as it was shown that S100B levels remain elevated even one year postpartum in women with a history of PE, compared to those with NP. This suggests, for the first time, that elevated blood S100B could potentially serve as a marker for persistent PE-associated CNS dysfunction. This finding is in accordance with previous epidemiological studies that showed an association between PE and neurodegenerative diseases [20,21,22,29,30], providing evidence that S100B could play a pivotal role as a long-term follow-up biomarker for CNS dysfunction in women surviving SPE or ECL.

#### 2.7.8. S100B’s Origin During Pregnancy

Both Wu et al. [50] and Andersson et al. [51] simultaneously examined the levels of S100B in maternal blood and other biological fluids, so as to elucidate its origin during pregnancy. In the study by Wu et al. [50], S100B was measured in maternal plasma, umbilical cord blood (UCB), and amniotic fluid. S100B was elevated in amniotic fluid but not in the UCB plasma of women with early SPE, demonstrating relatively low sensitivity (66%) and specificity (44%) for a cutoff value of 178 ng/mL. In the study by Andersson et al. [51], S100B, along with a panel of brain injury biomarkers including neurofilament light chain (NfL), tau protein, and neuron-specific enolase (NSE), was analyzed in the blood and cerebrospinal fluid (CSF) of women with SPE during the 3rd trimester. It was shown that S100B was increased in the sera of women with PE, but not in the CSF—in contrast to NfL, which was increased in both biological fluids. Moreover, the NfL concentration in maternal blood was correlated with its concentration in CSF, reflecting the CNS’s involvement in PE. The authors suggested that these findings might indicate a neuro-axonal injury in PE even when clinical manifestations and radiological findings are absent.

Friis et al. [52] confirmed the results of previous studies, as S100B was elevated in the maternal plasma of women with PE pregnancies. A group of non-pregnant women was also included in the study, and apart from S100B, additional biomarkers of CNS injury were investigated, including NfL, tau, and NSE. Comparative analysis revealed that only the NfL levels significantly differed between PE and non-pregnant women. In addition, using an in vitro blood–brain barrier (BBB) model, the study demonstrated that only plasma concentrations of NfL were associated with BBB alterations in PE. Thus, NfL was superior to S100B and a promising indicator for BBB dysfunction in PE.

Recently, the study by Busse et al. [38] investigated the S100B levels in maternal and UCB plasma, as well as in placental supernatant. Additionally, S100B expression was evaluated in maternal and UCB CD4^+^ T cells and CD19^+^ B cells in cases of spontaneous preterm birth (PTB) and in women who delivered following a PE or HELLP syndrome diagnosis, compared to term deliveries (TDs). It was found that the S100B concentrations were elevated in the maternal and UCB plasma of PTB and women with PE/HELLP, as well as in the UCB of small-for-gestational-age (SGA) infants. In maternal blood, S100B expression was upregulated in CD4^+^ T and CD19^+^ cells of PE/HELLP cases and mothers of SGA newborns, while in the UCB, S100B expression was elevated in CD19^+^ B cells of PTB, PE/HELLP, and SGA infants. Therefore, while S100B is primarily derived from the CNS, extra-cerebral sources, such as amniotic membranes, immune cells, and placenta trophoblasts, may also contribute to its increased levels, complicating the interpretation of elevated S100B levels in maternal blood [37,38,39].

## 3. Discussion

Preeclamptic CNS injury remains a significant challenge regarding its prediction, prognosis of severity, risk of progression to ECL, and long-term neurological impairments. In terms of pathophysiology, it represents a multifactorial process involving complex interactions among vascular dysfunction, inflammation, and oxidative stress [13,15]. These disruptions lead to a cascade of damage, including cerebral edema, BBB disruption, cerebral hemodynamic alterations, and endothelial dysfunction. These pathological changes are thought to precede the clinical onset of PE and are key contributors to its neurological complications, including ECL and seizures [14]. The early identification of biomarkers indicative of these processes is crucial for improving maternal outcomes, as timely intervention may help mitigate CNS-related complications and reduce the risk of long-term sequelae.

Once PE is established, clinical symptoms and signs often lack sufficient predictive value, as ECL can occur unexpectedly [53]. Additionally, imaging techniques, while valuable, are typically late indicators of cerebral involvement and are often impractical for routine use, especially in low-income countries where PE-related perinatal mortality is high [24,25,26,27,28,29,30]. The BBB disruption increases its permeability, allowing different types of molecules to leak from the CNS into maternal circulation [33]. Thus, brain injury biomarkers have become the focus of extensive research, with the dual aim of elucidating the extent and underlying mechanisms of cerebral involvement and serving as practical, cost-effective tools for clinical use. These biomarkers have the potential to provide critical information for the early diagnosis, monitoring, and optimal management of pregnancies complicated by PE, ultimately improving maternal and fetal outcomes. One of the most prominent brain injury biomarkers is S100B, which plays a neurotrophic and neuroprotective role under normal physiological conditions, while elevated levels in the bloodstream serve as an indicator of CNS injury, reflecting the damage to the BBB [33,34,35,36].

Based on the findings of the present systematic review, numerous studies have explored various aspects of alterations in S100B blood levels and its dynamic changes during pregnancy and postpartum [44,45,46,47,48,49,50,51,52,53]. These studies provide robust initial evidence supporting the predictive value of S100B, its correlation with the severity of PE, and its potential use as a long-term marker of CNS insult in PE pregnancies. In line with those studies, Hian Tan et al. [54] studied the protein cargo of the extracellular vesicles in plasma samples obtained from NP and PE women. The study found that these vesicles contained higher levels of S100B in PE women. However, no organ-specific origin of the above-mentioned extracellular vesicles was determined. Moreover, Bergman et al. [55] evaluated the levels of and dynamic changes in NfL and tau protein in the same study population previously examined by Wikström et al. [46], showing that both markers were increased at the end of the pregnancy in women developing PE, in contrast to healthy pregnancies. Plasma concentrations of tau protein and NfL, along with previously studied biomarkers such as S100B and NSE, were found to be elevated in women with PE compared to healthy pregnancies, suggesting early CNS involvement, even in mild-to-moderate cases. In a combined predictive model for PE, using all four biomarkers at different gestational ages, the model displayed an acceptable AUC for predicting PE’s occurrence at 25 weeks (0.77) and 28 weeks (0.75), with an excellent AUC in weeks 33 (0.89) and 37 (0.83). The median week for the diagnosis of PE was at 38 weeks, indicating that CNS involvement occurs months before the onset of clinical symptoms.

In summary, the utility of maternal blood S100B levels in women with suspected or established PE seems to be significant. S100B could serve not only as a potential predictive biomarker for the early detection of women at high risk for developing PE but also, from the early 3rd trimester, as a prognostic indicator of disease severity once PE is established. Moreover, S100B seems to be a promising biomarker for the long-term follow-up of PE encephalopathy, either independently or in combination with other brain injury biomarkers (such as NSE or NfL) and/or imaging techniques. Additionally, its combination with other biomarkers, pregnancy-related Doppler parameters, routine laboratory tests, and clinical manifestations of PE could contribute to the development of a comprehensive, multifactorial predictive model. This approach would enable more individualized risk stratification and timely therapeutic interventions, ultimately improving perinatal outcomes.

Nevertheless, based on the results of the present systematic review, there are still important aspects of S100B’s utility in PE to be investigated, reflecting the limitations of the existing evidence. In short, substantial heterogeneity was observed among the included studies regarding important aspects of research methodology and study characteristics, precluding the possibility of conducting a meaningful meta-analysis. Even though all studies adopted a case–control design, the characteristics of the study population differed extensively among the studies in terms of the type of PE investigated and maternal factors such as age, BMI, parity, sample type (plasma vs. serum) used, the timing of blood sampling during pregnancy, and the clinical parameters considered in statistical analysis—for example, the presence and nature of CNS-related symptoms and signs. In addition, the numbers of included studies and cases were relatively small, limiting our ability to draw comprehensive, broadly representative conclusions. Thus, statistical significance may be weakened, particularly when analyzing the association between S100B levels and different degrees of PE severity or specific clinical manifestations, such as HELLP syndrome or neurological symptoms and signs. This limitation may partially explain the inconsistency observed regarding the S100B levels observed in different studies. Furthermore, several studies reported incomplete data, including the precise S100B concentrations in different groups and the exact timing of sample collection during pregnancy, limiting the possibility of a statistical synthesis of the data extracted from similar groups of women with PE.

Importantly, the elevation of S100B in preeclamptic pregnancies might originate from extra-cerebral sources such as the amnion and lymphocytes, rather than being solely indicative of CNS release and, consequently, not directly correlated with neural damage [37,38,39]. This limitation might partially explain why, in combined models, using other specific brain injury biomarkers, NfL and tau demonstrated better predictive performance and correlation with BBB dysfunction [51,52]. However, these results should be confirmed by additional, larger case–control and cohort studies, as only limited data are available in the current literature.

However, S100B remains the most extensively studied biomarker of brain injury, not only in the context of preeclampsia but also across a broad range of neurological pathologies [32,33,34]. Future research should aim to further clarify the diagnostic and predictive value of S100B, especially at different stages of pregnancy, and in combination with other biomarkers (e.g., NfL, NSE). Thus, key objectives to address the current gaps in knowledge and enhance the clinical utility of S100B as a biomarker for PE are as follows: (a) To standardize measurement; establish a standardized methodology for measuring S100B in maternal blood, including the use of a specific sample type (plasma or serum) to ensure consistency and comparability across studies. (b) To evaluate S100B levels in different subtypes of PE; investigate S100B concentrations across various forms of PE, with a focus on determining specific predictive cutoff values for assessing the risk of developing PE and prognostic cutoff values for evaluating disease severity. (c) To evaluate the dynamic changes in S100B after the onset of PE; longitudinally examine the changes in S100B levels following the onset of PE using a repetitive sample collection methodology, correlating these trajectories with the progression of clinical symptoms and the presence of CNS-related manifestations. (d) To evaluate the predictive value regarding long-term CNS consequences; evaluate the predictive potential of S100B regarding the long-term neurological effects of SPE and ECL, establishing its role as a biomarker for monitoring CNS outcomes in affected women. (e) Finally, to compare and combine S100B with other specific brain injury biomarkers; combining S100B with other biomarkers seems to be a promising perspective that could provide an effective prognostic model for everyday clinical practice, aiming to avoid severe adverse neurological outcomes.

## 4. Conclusions

Preeclampsia is a serious multisystem disorder of pregnancy accompanied by a series of complications; the more severe ones include CNS. Thus, individualized monitoring of women with established PE is crucial for optimal perinatal care. S100B, a well-established biomarker of brain injury, has been shown to be elevated in the blood of women with PE compared to those with normal pregnancies. Notably, this increase is detectable from the early gestational weeks, preceding the onset of clinical symptoms. Additionally, higher S100B levels appear to be correlated with the severity of PE, including neural disturbances and the risk of HELLP syndrome, while postpartum levels may serve as a useful biomarker for the long-term monitoring of CNS injury associated with PE. Combination of S100B with other organ specific biomarkers could provide a predictive or prognostic model for the individualized care of women with PE. However, larger, high-quality studies are necessary in the future in order to establish a more comprehensive understanding of S100B’s utility in the context of PE.

## Figures and Tables

**Table 1:** Assessment of the quality and risk-of-bias estimation in the selected studies, using the Newcastle–Ottawa Scale (NOS).

Study Source	Reference Number	Year	Study Type	NOS Score	Quality
Schmidt, A. P., et al.	[44]	2004	Case–Control	7/9	High
Vettorazzi, J., et al.	[45]	2012	Case–Control	8/9	High
Wikström, A. K., et al.	[46]	2012	Observational Longitudinal Nested Case–Control	9/9	High
Bergman L., et al.	[47]	2014	Cross-Sectional Case–Control	8/9	High
Artunc-Ulkumen B., et al.	[48]	2015	Prospective Case–Control	7/9	High
Bergman L., et al.	[49]	2016	Longitudinal Case–Control	8/9	High
Wu, J., et al.	[50]	2021	Case–Control	6/9	Low
Andersson M., et al.	[51]	2021	Case–Control	8/9	High
Friis, T., et al.	[52]	2022	Observational Case–Control	7/9	High
Busse, M., et al.	[38]	2022	Cross-Sectional Case–Control	6/9	Low

**Table 2:** Study population and maternal/neonatal characteristics of the included studies.

	Definition	Study Population	Ethnicity	Maternal Age (Years)	BMI (Kg/m^2^)	Nullipara n (%)	Singleton n (%)	Smoking n (%)	MgSO_4_	Delivery (Weeks)	Birth Weight (g)
Schmidt, A. P. (2004) [44]	Former	18 PE	White 83%	24.8 ± 6.2	-	-	-	4	-	-	-
		11 HT	White 82%	32.4 ± 7.2	-	-	-	3	-	-	-
		10 ECL	White 80%	19.6 ± 6.9	-	-	-	2	-	-	-
		16 NP	White 69%	21.8 ± 4.8	-	-	-	4	-	-	-
Vettorazzi, J. (2012) [45]	Former	15 NP	-	24.5 ± 7.1	-	-	-	-	-	33.7 ± 4.7	2504 ± 909
		12 MPE	-	23.6 ± 7.6	-	-	-	-	-	36.7 ± 2.8	2975 ± 634
		34 SPE (8/34 HELLP) (5/34 ECL)	-	28.5 ± 9.3	-	-	-	-	In 8/34 at sampling	32.2 ± 3.8	1830 ± 851
Wikström, A. K. (2012) [46]	Former	37 NP	-	31 (28–33)	23 (21–25)	21 (60)	37 (100)	0	-	40.5 (40–41)	-
		16 PE	-	29 (26–32)	24 (22–28)	13 (83)	16 (100)	0	-	39.5 (38–41)	-
Bergman L. (2014) [47]	Former	53 PE	-	30 ± 5	27 ± 6	37 (70)	53 (100)	0 (0)	-	35.7 ± 4.1	2554 ± 988
		58 NP	-	30 ± 4	23 ± 3	29 (50)	58 (100)	2 (3)	-	40 ± 1.29	3658 ± 434
Artunc-Ulkumen B. (2015) [48]	Former	27 SPE (8/27 HELLP)	-	29.29 ± 4.29	30.39 ± 4.63	-	27 (100)	-	In all after sampling	33.6 ± 4.6	2140 ± 873
		36 NP	-	29.69 ± 4.85	29.65 ± 4.42	-	36 (100)	-	-	38.5 ± 1.4	3405 ± 407
Bergman L. (2016) [49]	Former	54 NP	-	30 ± 4	23 ± 5	29 (50)	54 (100)	2 (3)	-	40 ± 1.2	3658 ± 434
		48 PE	-	30 ± 5	27 ± 8	37 (70)	48 (100)	0 (0)	-	35.7 ± 4.1	2554 ± 988
Wu, J. (2021) [50]	Revised	9 early PE	Asian	35	18–25	9 (100%)	9 (100%)	-	-	-	FGR
		13 NP	Asian	35	18–25	13 (100%)	13 (100%)	-	-	-	Normal
Andersson M. (2021) [51]	Revised	15 SPE	-	32.5 (5.8)	27.5 (3.8)	12 (80)	-	1(7)	In 3/20	34.71 (4.04)	2200 (991)
		15 NP	-	31.9 (3.7)	22.9 (3.1)	5 (33)	-	1(7)	-	39.07 (5)	3430 (327)
Friis, T. (2022) [52]	Revised (all PE had proteinuria)	28 PE (16/28 SPE)	-	28 (25–32)	26 (23–29)	23 (82)	-	-	In 0/28	35 (25–41)	-
		28 NP	-	33 (29–35)	24 (22–26)	10 (36)	-	-	-	35 (27–38)	-
		16 non-pregnant	-	27 (24–36)	22 (20–25)	9 (56%)	-	-	-	-	-
Busse, M. (2022) [38]	Revised	17 TD (NP)	-	31.57 ± 5.56	-	-	-	-	-	38.76 ± 1.3	3362 ± 559
		17 PTB	-	28.83 ± 3.58	-	-	-	-	-	32.65 ± 3.02	1970 ± 524
		6 PE/HELLP	-	32.51 ± 6.79	-	-	-	-	-	30.33 ± 2.251	1181 ± 331

Revised definition of preeclampsia: new onset of hypertension after 20 weeks of gestation (systolic blood pressure ≥ 140 mmHg and/or diastolic blood pressure ≥ 90 mmHg), along with either utero-placental or maternal end-organ dysfunction. Former definition of preeclampsia: new onset of hypertension after 20 weeks of gestation (systolic blood pressure ≥ 140 mmHg and/or diastolic blood pressure ≥ 90 mmHg), along with proteinuria (≥2 or more on a dipstick or ≥300 mg in a 24 h urine sample). BMI: body mass index, PE: preeclampsia, MPE: mild preeclampsia, SPE: severe preeclampsia, HT: chronic hypertension, ECL: eclampsia, MgSO_4_: magnesium sulfate, NP: normal pregnancy, FGR: fetal growth restriction, TD: term delivery via planned caesarean section (normal pregnancies), PTB: preterm birth via planned caesarean section, HELLP: hemolysis, elevated liver enzymes (transaminases), and low platelet count.

**Table 3:** Symptoms, signs, or laboratory findings of severe preeclampsia.

	Study Population	SBP (mmHg)	DBP (mmHg)	Proteinuria (>5 g/24 h)	Premonitory Symptoms	Tendon Reflexes	Abdominal Pain	Low PTL	Hepatic Enzymes	Pulmonary Edema	Fetal Distress
Schmidt, A. P. (2004) [44]	18 PE	-	-	-	-	-	-	-	-	-	-
	11 HT	-	-	-	-	-	-	-	-	-	-
	10 ECL	160.0 ± 18.0	102.2 ± 18.6	-	-	-	-	-	-	-	-
	16 NP	-	-	-	-	-	-	-	-	-	-
Vettorazzi, J. (2012) [45]	15 NP	-	-	-	-	-	-	-	-	-	-
	12 MPE	-	-	-	-	-	-	-	-	-	-
	34 SPE (8/34 HELLP) (5/34 ECL)	165.5 ± 23.0	105.9 ± 16.7	16 (47.1)	18 (53%)	-	1 (2.9%)	13 (37.1%)	7 (20.6%)	1 (2.9%)	6 (17.6%)
Wikström, A. K. (2012) [46]	37 NP	-	-	-	-	-	-	-	-	-	-
	16 PE	-	-	-	-	-	-	-	-	-	-
Bergman L. (2014) [47]	53 PE	-	-	-	-	-	-	-	-	-	-
	58 NP	-	-	-	-	-	-	-	-	-	-
Artunc-Ulkumen B. (2015) [48]	27 SPE (8/27 HELLP)	171.1 ± 20.8	102.5 ± 14.2	-	-	-	-	-	-	-	5
	36 NP	-	-	-	-	-	-	-	-	-	-
Bergman L. (2016) [49]	54 NP	-	-	-	-	-	-	-	-	-	-
	48 PE	-	-	-	-	-	-	-	-	-	-
Wu, J. (2021) [50]	9 early PE	160–190	100–120	-	-	-	-	-	-	-	PBI FGR NST
	13 NP	-	-	-	-	-	-	-	-	-	-
Andersson M. (2021) [51]	15 SPE	-	-	-	9 (60%)	2 (13%)	1 (7)	3 (20%)	5 (33%)	-	-
	15 NP	-	-	-	-	-	-	-	-	-	-
Friis, T. (2022) [52]	28 PE (16/28 SPE)	-	-	-	10 (36%)	-	-	-	-	-	-
	28 NP	-	-	-	-	-	-	-	-	-	-
	16 non-pregnant	-	-	-	-	-	-	-	-	-	-
Busse, M. (2022) [38]	17 TD (NP)	-	-	-	-		-	-	-	-	-
	17 PTB	-	-	-	-		-	-	-	-	-
	6 PE/HELLP	-	-	-	-		-	6	6	-	-

Premonitory symptoms: blurred vision, diplopia, scintillating scotomas, cephalalgia. Abdominal pain: persistent epigastric/upper right quadrant abdominal pain. Low PTL: thrombocytopenia (<100.000 platelets/μL). Increased hepatic enzymes: at least twice the reference values of alanine aminotransferase (ALT) or aspartate aminotransferase (AST). Fetal distress: including perinatal brain injury (PBI), non-reassuring (abnormal) non-stress test (NST), fetal growth restriction (FGR), defined either as birth weight >2 SDs below mean birth weight for gestational age of the reference population, or as estimated by fetal weight < 10% with concomitant oligohydramnios or abnormal Doppler ultrasound of the umbilical vessels. PE: preeclampsia, MPE: mild preeclampsia, SPE: severe preeclampsia, HT: chronic hypertension, ECL: eclampsia, NP: normal pregnancy, FGR: fetal growth restriction, TD: term delivery via planned caesarean section (normal pregnancies), PTB: preterm birth via planned caesarean section, HELLP: hemolysis, elevated liver enzymes (transaminases), and low platelet count (PTL).

**Table 4:** Laboratory parameters, blood levels of S100B, and statistical results of the included studies.

Author (Year)	Sample Type	Method	Study Population	Sampling Time	S100B Levels	*p*	Sensitivity–Specificity–Predictive Value
Schmidt, A. P. (2004) [44]	Serum	Sangtec 100, ELISA, Diasorin, MN (µg/L)	18 PE	34.7 ± 4.1 weeks	0.185 (0.14)	<0.05 Between ECL and all other groups	-
			11 HT	36.8 ± 1.9 weeks	0.186 (0.12)		
			10 ECL	33.5 ± 3.9 weeks	0.424 (0.194)		
			16 NP	34.6 ± 6.4 weeks	0.147 (0.07)		
Vettorazzi, J. (2012) [45]	Serum	Luminescence assay (LIAmat Sangtec 100, Sweden) (µg/L)	15 NP	-	0.04 ± 0.05	-	-
			12 MPE	-	0.07 ± 0.05	-	
			34 SPE (8/34 HELLP) (5/34 ECL)	-	0.2 0 ± 0.19	0.002 to NP 0.003 to MPE	
Wikström, A. K. (2012) [46]	Plasma	Sangtec 100 ELISA, Diasorin, MN (µg/L)	37 NP	10, 25, 28, 33, 37 weeks	0.047–0.052	0.71 within group <0.05 at 33 and 37 week vs. PE group	-
			16 PE		0.052–0.075	<0.05 within group	
Bergman L. (2014) [47]	Plasma	Sangtec 100 ELISA, Diasorin, MN (µg/L)	53 PE	35.2 ± 4.4 weeks	0.12 (0.02–0.77)	<0.001	Cutoff: 0.14 µg/L Sensitivity: 44% Specificity: 86% AUC: 0.71
			58 NP	34.5 ± 4.8 weeks	0.07 (0.02–0.31)		
Artunc-Ulkumen B. (2015) [48]	Serum	Bio Vendor ELISA (Cobas e 411, Roche) (µg/L)	27 SPE (8/27 HELLP)	-	0.13 ± 0.01	0.025	Cutoff: 0.0975 µg/L Sensitivity: 81.4% Specificity: 58.3% AUC: 0.712 PPV: 59.45% NPV: 80.7
			36 NP	-	0.09 ± 0.05		
Bergman L. (2016) [49]	Plasma	Sangtec 100 ELISA, Diasorin, MN (µg/L)	53 NP	398 ± 36 days post delivery	0.06 (0.04–0.07)	<0.05	-
			58 PE	406 ± 40 days post delivery	0.07 (0.06–0.09)		
Wu, J. (2021) [50]	Plasma	ELISA ng/mL SPRi ng/mL	9 early PE	32–34 weeks	198.91 ± 51.02 209.01 ± 27.54	0.007 for ELISA <0.001 for SPRi	>178 ng/mL (ELISA) >181 ng/mL (SPRi) Sens: 66/100% (ELISA/SPRi) Spec:44/84% (ELISA/SPRi)
		ELISA ng/mL SPRi ng/mL	13 NP		111.63 ± 42.64 115.18 ± 51.02		
Andersson M. (2021) [51]	Serum	Cobas Elecsys platform (µg/L)	15 PE	243 days	0.08 (0.06–0.1)	<0.01	-
			15 NP	273.5 days	0.05 (0.04–0.06)		
Friis, T. (2022) [52]	Plasma	Sangtec 100 ELISA, Diasorin, MN (µg/L)	28 PE (SPE16/28)	35 (29–37) weeks	0.08 IQR 0.05–0.10	<0.01	-
			28 NP	35 (27–38) weeks	0.05 IQR 0.03–0.08		
			16 non-pregnant	-	-	-	-
Busse, M. (2022) [38]	Plasma	ELISA R&D systems USA (pg/mL)	17 TD (NP)	38.76 ± 1.3 weeks	-	TD vs. PTB: <0.001 TD vs. PE/HELLP: 0.009	-
			17 PTB	32.65 ± 3.02 weeks	-		
			6 PE/HELLP	30.33 ± 2.251 weeks	-		-

PE: preeclampsia, MPE: mild preeclampsia, SPE: severe preeclampsia, HT: chronic hypertension, ECL: eclampsia, NP: normal pregnancy, SPRi: surface plasmon resonance imaging, AUC: area under the curve, TD: term delivery via planned caesarean section (normal pregnancies), PTB: preterm birth via planned caesarean section, HELLP: hemolysis, elevated liver enzymes (transaminases), and low platelet count (PTL).

**Table 5:** Conclusions of the included studies.

Study	Conclusions
Schmidt, A. P. (2004) [44]	Increased level of S100B in maternal serum is likely to be associated with eclampsia.
Vettorazzi, J., (2012) [45]	Elevated serum S100B levels in pregnant women with SPE suggest some kind of neural damage and subsequent astrocytic release of S100B. As no difference was detected between women with SPE and those with eclampsia, S100B changes were not dependent on the progression from severe preeclampsia to eclampsia.
Wikström, A. K. (2012) [46]	Levels of S100B in maternal plasma are increased during pregnancy in women who develop preeclampsia compared to healthy pregnancies, and the levels of S100B increase several weeks before clinical symptoms of the disease appear.
Bergman L. (2014) [47]	Plasma levels of S100B are elevated among women with preeclampsia compared with control subjects; furthermore, they increase irrespective of blood pressure level, showing association with visual disturbances, which might reflect possible CNS effects.
Artunc-Ulkumen B. (2015) [48]	Serum S100B levels may be a potential marker in severe preeclampsia for the severity of hypoperfusion in the brain and placenta, as well as the subsequent risk of organ failure.
Bergman L. (2016) [49]	The levels of NSE and S100B are significantly elevated in pregnant women with preeclampsia compared to those with normotensive pregnancies and persist up to one year postpartum, suggesting potential long-term neurological implications in individuals with a history of preeclampsia.
Wu, J. (2021) [50]	Increased levels of S100B in maternal plasma and amniotic fluid in early-onset SPE, but not in the cord blood (CB) plasma. There is a positive correlation in S100B concentration between maternal plasma and amniotic fluid. SPRi-S100B is more sensitive to ELISA-S100B for the diagnosis of early-onset SPE.
Andersson M. (2021) [51]	Women with preeclampsia demonstrated increased serum and plasma concentrations of S100B and NfL, respectively. Concentrations of NfL, but not S100B, were increased in CSF compared to women with normal pregnancies. Neurofilament light chain emerged as a promising circulating cerebral biomarker in preeclampsia.
Friis, T. (2022) [52]	Increased circulating concentrations of S100B, NfL, tau, and NSE were present in the maternal plasma of women developing SPE. Concentrations of NfL were also higher in women with PE compared with non-pregnant women. NfL could be a promising biomarker for BBB alterations in preeclampsia.
Busse, M. (2022) [38]	Εnhanced S100B concentration in maternal and CB plasma in women with preterm birth (PTB) and women with preterm delivery following PE/HELLP diagnosis, compared to women with term delivery (TD). S100B was positively correlated with interleukin-6 (IL-6) levels. S100B expression was enhanced in inflammatory events associated with PTB.

PE: preeclampsia, SPE: severe preeclampsia, SPRi: surface plasmon resonance imaging, CNS, NSE: neuron-specific enolase, CSF: cerebrospinal fluid, NfL: neurofilament light chain, BBB: blood–brain barrier, CB: cord blood, TD: term delivery via planned caesarean section (normal pregnancies), PTB: preterm birth via planned caesarean section, HELLP: hemolysis, elevated liver enzymes (transaminases), and low platelet count (PLT).

## Data Availability

Not applicable.

## Supplementary Materials

The following supporting information can be downloaded at: https://www.mdpi.com/article/10.3390/biom15060840/s1, File S1: S1. PRISMA 2020 checklist.

## Appendix A

**Table A1:** Newcastle–Ottawa Quality Assessment Scale (NOS) for Case–Control Studies, applied in each study.

Study Source	Study Type	NOS Score	Remarks
Schmidt, A. P., et al. (2004) [44]	Case–Control Study	7/8	The study has strong participant criteria, thorough data collection, and robust stats, but limitations include blinding and controlling for potential confounders.
Vettorazzi, J., et al. (2012) [45]	Case–Control Study	8/9	Reasonable methodology in participant selection, group comparability, and outcome assessment, but enhancing representativeness and addressing key confounding factors would add value.
Wikstrom, A. K., et al. (2012) [46]	Observational Longitudinal Nested Case–Control Study	9/9	High methodological quality in participant selection, comparability, exposure, and outcome assessment. A larger sample could enhance the results’ robustness and generalizability.
Bergman L., et al. (2014) [47]	Cross-Sectional Case–Control Study	8/9	Meets the NOS criteria for group selection, comparability, and exposure/outcome assessment effectively, but potential confounding factors remain.
Artunc-Ulkumen B., et al. (2015) [48]	Prospective Case–Control Study	7/9	The study addresses the NOS criteria regarding participant selection and exposure/outcome measurement, but potential bias in control recruitment and unaddressed confounding factors are concerns.
Bergman L., et al. (2016) [49]	Longitudinal Case–Control Study	8/9	High quality of study design, methodology, and reporting. Lack of blinding.
Wu, J., et al. (2021) [50]	Case–Control Study	6/9	Moderate quality, unclear control ascertainment, and small sample size.
Andersson M., et al. (2021) [51]	Case–Control Study	8/9	The study effectively meets the NOS criteria for group selection and exposure/outcome assessment but lacks detail on controlling for confounding variables.
Busse, M., et al. (2022) [38]	Cross-Sectional Case–Control Study	6/9	Clear criteria, diverse biomarkers, and S100B–immune cell correlations explored. Limitations: small sample, bias, incomplete data, long storage, and no multiple comparison adjustments, affecting validity.
Friis, T., et al. (2022) [52]	Observational Case–control study	7/9	The study shows strong design and methodology, but considerable limitations in sample size and potential confounders when interpreting the findings.
